# Commissioning and clinical implementation of an MLC tracking system: An evaluation of AAPM TG‐264 guidelines

**DOI:** 10.1002/acm2.70492

**Published:** 2026-03-03

**Authors:** André Haraldsson, Tobias Pommer, Emilia Persson, Hedda Enocsson, Mustafa Kadhim, Adalsteinn Gunnlaugsson, Per Munck af Rosenschöld

**Affiliations:** ^1^ Radiation Physics, Department of Hematology, Oncology & Radiation Physics Skåne University Hospital Lund Sweden; ^2^ Medical Radiation Physics, Department of Clinical Sciences Lund University Lund Sweden; ^3^ Medical Radiation Physics, Department of Translational Medicine Lund University Malmö Sweden; ^4^ Department of Hematology, Oncology & Radiation Physics Skåne University Hospital Lund Sweden

**Keywords:** radiotherapy

## Abstract

**Background:**

Precise radiotherapy relies on accurately targeting tumours while minimising exposure to healthy tissue, yet patient and organ motion complicate treatment delivery. To address intra‐fractional motion, multi‐leaf collimator (MLC) tracking systems have recently been adopted, adapting beam shapes in real‐time. The American Association of Physicists in Medicine (AAPM) Task Group 264 (TG‐264) provides guidelines for safely commissioning such tracking systems, yet these guidelines were initially developed for conventional linear accelerators and require evaluation, especially for newer platforms such as Radixact Synchrony.

**Purpose:**

This study aimed to: (i) evaluate the clinical performance and dosimetric accuracy of the Radixact Synchrony MLC tracking system according to AAPM TG‐264 guidelines, from commissioning to clinical implementation; and (ii) critically assess and suggest practical refinements to these guidelines based on experiences with this novel tracking technology.

**Methods:**

Commissioning followed TG‐264 recommendations, adapted for Radixact Synchrony, utilizing three tracking modes: fiducial‐based, markerless adaptive, and marker‐based adaptive tracking. Performance was assessed with multiple test systems, including the Delta4 Phantom+, HexaMotion, Quasar platform, and film dosimetry. Measurements included geometric accuracy of phantom trace tracking, dosimetric accuracy of delivered dose to movable phantom, and system latency. Clinical protocols established treatment planning, quality assurance (QA), safety procedures, and clinical decision pathways, focusing on prostate and lung cancer treatments.

**Results:**

The Synchrony system demonstrated substantial improvements in geometric accuracy compared to non‐MLC‐tracking approaches. Fiducial‐based tracking achieved a root mean square error (RMSE) of 0.76 ± 0.27 mm compared to 3.99 ± 2.84 mm without tracking (*p* = 0.008), with a mean absolute error (MAE) reduction to 0.36 ± 0.12 mm. Markerless adaptive tracking resulted in similar accuracy (RMSE 0.80 ± 0.15 mm, MAE 0.68 ± 0.15 mm). Dosimetric evaluations revealed consistent improvements, with gamma pass rate ≥ 95% (criteria 2%/2 mm) for tracked plans, significantly outperforming static plans under dynamic conditions (*V* = 7.0, *p* = .037). System latency was measured one time at approximately 630 ms for fiducial tracking without external breathing monitoring, slightly exceeding TG‐264′s ideal threshold (500 ms), yet well within the manufacturer's tolerance (1.5 s). Clinical cases confirmed feasibility, showing median deviations of 2.0–3.9 mm for prostate tracking and around 3.3 mm for markerless lung tracking. Safety protocols and clinical pathways developed during implementation ensured treatment robustness.

**Conclusions:**

The Radixact Synchrony MLC tracking system successfully met TG‐264 guidelines, significantly improving geometric and dosimetric accuracy during real‐time tumour tracking. However, practical implementation highlighted necessary adaptations to TG‐264 recommendations for non‐standard platforms such as Radixact, specifically regarding QA protocols, latency tolerance, and handling of the system's unique characteristics (pneumatic MLC, jaw tracking, and flattening‐filter‐free beams). Our findings underscore the importance of maintaining conservative margins initially, rigorous QA, specialized staff training, and careful patient selection strategies. Further clinical trials focusing on safe margin reduction strategies are essential for optimizing the clinical benefits of advanced tracking technologies.

## 1 INTRODUCTION

Radiotherapy remains one of the cornerstones in cancer treatment, with its success heavily dependent on the precision of dose delivery to the target while sparing healthy tissue. However, patient and organ motion, both between and during treatment sessions, pose significant challenges. These uncertainties are broadly categorized into inter‐fractional and intra‐fractional movements. While inter‐fractional motion can often be accounted for during patient setup, intra‐fractional motion may be unpredictable and can lead to significant deviations in dose deposition if not properly managed. Increased treatment margins are commonly employed to mitigate this risk, but they come at the expense of irradiating larger volumes of healthy tissue, potentially raising the incidence and severity of toxicities. Furthermore, large movements during treatment can cause underdosing of the target, compromising the likelihood of tumour control.^1^, ^2^, ^3^

To address challenges with intra‐fraction motion, several techniques for motion management have been introduced, including ultrasound tracking, kilovoltage (kV) imaging, and more recently, multi‐leaf collimator (MLC) tracking. MLC tracking enables real‐time adaptation to patient motion by adjusting the beam shape dynamically. This approach relies on accurate tumour position detection through kV imaging and a responsive, low‐latency mechanism, typically using MLCs or jaws, to conform the radiation beam. Although MLC tracking has been thoroughly explored on conventional C‐arm linear accelerators^4^ its recent application on the Radixact system (Accuray, Madison, USA) under the commercial name “Synchrony” has introduced new clinical possibilities as well as challenges. The Synchrony system, already well‐established on the CyberKnife (Accuray, Madison, USA) platform, and, more recently, on the Radixact,^5^, ^6^, ^7^, ^8^, ^9^, ^10^, ^11^ now supports both prostate and lung cancer treatments through real‐time imaging and motion compensation.

Prostate cancer treatments require high precision since significant intra‐fractional motion may occur due to organ and patient movement.^12^, ^13^, ^14^ Fiducial markers are commonly implanted to aid in the accurate localization of the prostate immediately before and during treatment. Similarly, lung cancer treatments to smaller lesions, often delivered in hypofractionated regimens, benefit from reducing margins by tracking respiratory‐induced motion. In these contexts, the ability to follow and adjust for target motion is critical.^15^, ^16^

A central framework guiding the safe implementation of these advanced tracking systems is provided by the American Association of Physicists in Medicine (AAPM) Task Group 264 (TG‐264) report.^17^ This document outlines comprehensive guidelines for commissioning real‐time motion tracking systems, including specifications for system accuracy, quality assurance (QA), treatment planning, and delivery. In our study, we leverage the TG‐264 recommendations to assess the performance of an MLC tracking system integrated on the Radixact platform. Our dual objectives were: (i) to evaluate the clinical performance of the MLC tracking system—from acceptance testing through commissioning and clinical deployment—and (ii) to provide feedback on the TG‐264 guidelines based on our practical experience implementing the technology.

This paper details the systematic evaluation of the Synchrony system using a combination of static and dynamic tests on phantoms and clinical data. We compare the system's performance with and without tracking, assess its dosimetric accuracy, and examine the overall impact on treatment delivery. Moreover, as a first, we discuss challenges encountered when applying TG‐264 recommendations overall, and specifically to a system with unique characteristics such as a pneumatic MLC, jaw tracking for longitudinal motion, and a flattening filter‐free (FFF) beam. By sharing our experience, we aim to contribute to the refinement of TG‐264, the development of AAPM guidelines, and offer insights for centers implementing tracking technologies.

## 2 MATERIALS AND METHODS

Our methodology was designed to follow the TG‐264 guidelines while adapting them to the specific technical aspects of the Radixact Synchrony system. We outline below the details of system specific settings, measurement systems, clinical implementation guidelines, clinical decision pathways, quality assurance program, measurement and data analysis, and statistical analyses.

### 2.1 System specific settings for MLC tracking

The Synchrony system integrates multiple MLC tracking modes that allow for real‐time adjustment of the treatment beam. It is equipped with an orthogonal kV imaging source and detector system that can capture up to six images per gantry rotation. An infrared (IR) diode array further augments the system by monitoring the patient's breathing amplitude. The Synchrony system combines internal kV image‐based localization with external respiratory monitoring to model target motion in real time. Orthogonal kV images provide the 3D position of fiducials or tumour/markers, while an IR diode array records the patient's breathing signal. A correlation model links the external surrogate to the internal target position and is updated when new kV images are acquired. A prediction model then compensates for system latency by forecasting the target position slightly ahead in time, allowing MLC leaves and jaws to dynamically adjust the beam aperture so it continuously follows the moving target during treatment. The system can operate in three distinct modes:

1. **Fiducial tracking**:
  This mode relies on implanted markers to determine tumour position using kV imaging. It's a reactive mode as the beam is shifted towards the measured target position after each kV image and is used for non‐respiratory motion. The system uses several thresholds (denoted T1–T5) to govern tracking accuracy:
  ∘ **T1 (Potential difference)**: A statistical estimate of the 3D error for the model's prediction.
  ∘ **T2 (Rigid body)**: The maximum change in the pairwise distance between fiducials, comparing the planned and current radiographic positions.
  ∘ **T3 (Target offset)**: The discrepancy between the planned and current 3D target position.
  ∘ **T4 (Auto pause delay)**: The time delay before an automatic treatment pause, triggered when a kV image is processed.
  ∘ **T5 (Target outside jaw range threshold)**: The maximum allowable time the target may be partially outside the jaw range before beam interruption.
2. **Markerless adaptive lung tracking**:
  In this mode, an adaptive model is employed to correlate the patient's respiratory motion with tumour movement. The system identifies the tumour based on its density and shape relative to the surrounding tissue. Here, the thresholds T1, T3, T4, and T5 are applicable, while T2 is omitted due to the absence of fiducials. An additional parameter, **T6 (Measured Delta)**, is used to quantify the difference between the predicted and actual target positions on each KV‐image.
3. **Marker‐based adaptive tracking**:
  This mode is a hybrid that combines the advantages of both fiducial and adaptive tracking, using implanted markers to track the tumour for the motion model with improved accuracy. The thresholds are similar to markerless adaptive, with the added **rigid body** threshold for marker distance.

The reported Synchrony results were obtained from the tracking output used for beam adaptation in each mode. For markerless (lung) adaptive tracking, this output is based on a combination of the correlation model and the prediction model (the prediction is used to compensate latency). In contrast, fiducial (prostate) tracking is reactive and does not use a prediction model; the aperture is updated based on the most recent kV‐derived position after each image.

### 2.2 Measurement systems

To test and commission the MLC tracking system, a variety of measurement platforms were employed. The Delta4 Phantom+ (ScandiDos, Uppsala, Sweden) served as the primary dosimetric tool, while the HexaMotion system (ScandiDos, Uppsala, Sweden) provided a five‐dimensional motion platform capable of simulating translational and rotational target motion, as well as independent breathing surrogate movements. Traces corresponding to both sinusoidal and patient‐specific motions were generated and imported into this platform for testing. Film dosimetry was performed using the Quasar Platform (Modus Medical, Ontario, Canada), which offers one‐dimensional motion, in combination with EBT3 radiochromic film (Ashland Advanced Materials, Bridgewater, USA). The films were scanned with an EPSON 4990 flatbed scanner and analyzed using FILMQAPro software, enabling high spatial‐resolution dosimetry. In addition, an anthropomorphic phantom (PBU‐60, Kyoto Kagaku, Japan) with a moving chest target and surrogate motion platform was used to perform end‐to‐end testing in a clinically realistic setup.

### 2.3 Clinical implementation guidelines

Following TG‐264 recommendations, our clinical implementation protocol was divided into several key components, outlined in the subsections below:

#### 2.3.1 Treatment planning evaluation

Treatment planning for both fiducial‐based and markerless tracking involved a thorough evaluation of allowed motion ranges, machine limitations (MLC and jaw travel), and the differential motion between the target and organs at risk (OARs). For fiducial tracking, synthetic CT (sCT) generated from planning MRI, is routinely used for hypofractionated prostate treatments in our clinic, were used and compared to conventional CT images using previously validated patient dataset^18^ Individual beam gating tolerances were defined based on clinical discussion and method evaluations for maximum target offset (T3). For markerless tracking, 4D‐CT was used to evaluate the temporal aspects of respiratory motion, where mid‐position planning was used to address limitations in jaw tracking for cranio‐caudal motion. Since the jaw range is ± 12.5 mm or ± 20.0 mm in the cranial‐caudal direction, we combined mid‐position planning and online registration on soft tissue and bone close to the tumour to center the movement range of the tumour during treatment.

#### 2.3.2 Treatment planning

Treatment plans were developed using both Precision planning software 3.0 (Accuray Inc., Madison, USA) and RayStation 12B (RaySearch, Stockholm, Sweden). Each plan used six distinct kV imaging angles to assess 3D motion at delivery. The pitch and modulation factor were balanced for optimal gantry period, OAR doses to dose coverage, and imaging interval. RayStation plans were generated with a higher pitch than Precision to achieve a comparable gantry period (and thus kV imaging interval) and similar plan modulation. Treatment plans for testing movement, end‐to‐end and latency were optimized directly on the Delta4 phantom, and film measurement plans directly on the Quasar phantom. A smaller set of plans was planned on patient geometries and recalculated and measured on the Delta4 phantom. For testing purposes, a circular planning target volume (PTV) was created on the Delta4 planning CT, along with adjacent OAR structures simulating rectum or bladder. Plans for end‐to‐end testing were also optimized on MRI‐only datasets of patients, to verify the consistency between synthetic and conventional CT planning, Table 1. Plan complexity was assessed from the modulation factor (MF). For clinical prostate plans, a 7 mm margin is employed as clinical target volume (CTV) to PTV margin.

**TABLE 1:** Plan parameters used for tracking and stationary measurements. *Pitch* = field overlap per field width and gantry rotation, *MF* = Modulation Factor, *FW* = Field Width.

Plan type	Delivery time (s)	Fraction dose (Gy)	Imaging angles (°)	Gantry period (s)	Pitch	MF	FW
Precision	264	2.0	25, 75, 134, 201, 250, 306	18.1	0.20	1.6	2.5
RayStation	156	2.0	25, 75, 134, 201, 250, 306	17.6	0.43	1.6	2.5
End‐to‐end	391	6.1	20,80,140,200,260,310	26.3	0.25	1.7	2.5
MRI‐only	505	6.1	20,80,140,200,260,310	28.4	0.20	1.9	2.5

#### 2.3.3 Treatment delivery for MLC tracking

The evaluation of treatment delivery focused on verifying that real‐time motion tracking was correctly synchronized with dose delivery. First, connectivity and communication between all subsystems were tested under both static and dynamic conditions. The system's self‐checks for detecting motion outside tolerance limits were confirmed by deliberately introducing offsets into motion traces. Dose accumulation was evaluated both on phantoms and recalculated patient datasets to ensure that target coverage was preserved under motion. To link planning with delivery, routines for daily imaging were implemented to compare 4D‐CT planning geometries with 3D daily CT images. Finally, delivery accuracy was tested using both sinusoidal and patient‐specific motion traces and comparing measured doses with planned doses calculated on the HexaMotion/Delta4 and Quasar platforms, thereby validating performance across both simple and clinically realistic motion scenarios.

#### 2.3.4 Minimum requirements for a real‐time target position monitoring system

According to TG‐264, a robust real‐time position monitoring system must meet several criteria. First, the surrogate position signal should be accurate to within 1 mm, with an uncertainty below 2 mm. In our study, this was verified by comparing the Synchrony system's recorded signal to the motion traces on the HexaMotion platform. Second, the system should capture surrogate signals at a frequency exceeding 3 Hz for respiratory motion; we confirmed this by analyzing log file and compared them against the programmed motion traces. Third, the overall system latency, from image acquisition to mechanical jaw response, should ideally be under 500 ms. We assessed this by extracting timestamps from system log files and calculating the delay between the start of kV image acquisition and the recorded end of jaw motion which were both recorded in the same log file. Specifically, system latency was defined as the elapsed time from **kV image acquisition start** to **completion of longitudinal jaw motion**. Particularly, we used the timestamp of the **anode current peak in the kV generator** (proxy for x‐ray pulse / image acquisition), and the timestamp corresponding to **jaw motion end** in the longitudinal (IEC‐Z / SI) direction. Finally, TG‐264 specifies that a comprehensive QA program must be in place. In our implementation, this was verified through a combination of geometric accuracy tests using the Delta4/HexaMotion setup, dosimetric accuracy using the Delta4 3D‐diode array and Quasar film measurements, and periodic QA checks to ensure both hardware and software reliability.

#### 2.3.5 Safe implementation and limitations of MLC tracking

Safe implementation strategies were developed in accordance with TG‐264. Specific considerations included:

- Ensuring that treatment margins were not reduced prematurely, given the uncertainties inherent in dynamic tracking.
- Evaluating the impact of residual geometric errors on dosimetry.
- Addressing the limitations imposed by the binary nature of the pneumatic MLC and the compensatory mechanisms for jaw tracking.
- Establishing fallback procedures and action thresholds in the event of unexpected system behavior.

Moreover, our clinical protocol required a thorough training program for radiation therapists, nurses, and medical physicists. Site‐specific decision algorithms were developed to guide the use of tracking, particularly in cases where targets approached critical structures or where residual uncertainties might compromise treatment quality.

### 2.4 Clinical decision pathways

To ensure that MLC tracking was applied where it provided the greatest clinical benefit, patient selection followed structured decision pathways. For prostate cancer, patients were included if they had high‐risk disease, were eligible for fiducial implantation, and presented with anatomical conditions compatible with MRI‐only simulation. For lung cancer, eligibility required a clearly distinguishable lesion on CT and the ability to maintain reproducible respiratory motion during simulation. Beyond eligibility, the decision to use tracking was guided by the expected dosimetric gain—particularly when the target was located close to critical structures where motion could otherwise compromise OAR sparing. Safety considerations were equally important: tracking was only implemented when residual uncertainties were judged unlikely to cause clinically significant dose deviations. To support this decision process, all treatment plans were optimized on patient geometry, recalculated on the Delta4 phantom, and measured as QA plans to confirm deliverability and dosimetric robustness prior to clinical use.

### 2.5 Quality assurance program

A QA program is critical to maintaining safe and effective treatment delivery. Our QA strategy was implemented in two stages:

1. **System QA**: This included baseline tests for response times, anomalous condition detection, and end‐to‐end dosimetry validation. We performed both static and dynamic tests to ensure the system could reliably detect and compensate for motion.
2. **Patient‐specific QA**: Using TG‐264 guidelines, patient‐specific QA tests were conducted with criteria such as a gamma pass rate (GPR) of 95% using 3% dose difference and 2 mm distance‐to‐agreement (DTA) and 15% threshold with global gamma. Additional offset tests were included to verify that the imaging and registration processes were robust even with deliberate perturbations.

The QA program also encompassed periodic recalibration, staff retraining, and documentation updates to capture any deviations from the established protocols.

### 2.6 Measurements and data acquisition

Comprehensive measurements were performed primarily on the Delta4 phantom combined with the HexaMotion motion platform, supplemented by the Quasar and anthropomorphic phantoms for specific tests. To establish baseline performance, static plans were delivered to the stationary Delta4 phantom, which allowed us to verify dose calculation accuracy in the absence of motion. Dynamic testing was then performed on the Delta4/HexaMotion setup using nine clinical prostate motion traces and additional traces with deliberate offsets. These traces were adapted from the LMU dataset (20 ms resolution) and programmed into the HexaMotion platform, ensuring physiologically realistic velocities (1–3 mm/s) and accelerations (6–14 mm/s^2^).

The system's motion model was evaluated by comparing its predicted 3D error estimate (“potential difference”) against the actual deviations measured on the Delta4 detector array during motion. Geometric accuracy was quantified using mean absolute error (MAE) and root mean square error (RMSE), with 95% confidence intervals reported as margins of error. Specifically, RMSE when calculated between motion model and phantom trace, was defined as the 3D radial RMSE representing residual tracking error.

For markerless adaptive tracking, measurements were extended to the anthropomorphic PBU‐60 phantom. Both sinusoidal traces and patient‐specific respiratory traces (derived from 4D CT scans of lung cancer patients) were programmed into the phantom's moving chest target with peak‐to‐peak amplitudes of *y* = 20.0 mm, *z* = 10.0 mm, and *y* = 10.7 mm, *z* = 8.9 mm, respectively. The programmed motion was then compared directly with the Synchrony system's assessed motion trajectory, providing an independent check of the system's performance under realistic clinical breathing patterns. In addition, high‐resolution film measurements on the Quasar platform were used to validate dosimetric accuracy during one‐dimensional programmed motion. Further, the patient 3D trace (derived from 4D CT), with and without an added 5 mm baseline drift, and artificial sin y + sin z traces were measured with the Hexamotion and Delta4 setup.

### 2.7 Statistical analysis

Statistical significance was determined using Student's t‐test for normally distributed data and the Mann–Whitney rank‐sum test or Wilcoxon signed rank test for nonparametric data. Accuracy metrics were reported as MAE and RMSE. We defined the margin of error as the value corresponding to the 95th percentile of the absolute deviations (i.e. 95% of all deviations were smaller than this value). Correlation analyses between the potential difference from the model and the actual 3D displacement were performed using Pearson or Spearman correlation coefficients, where applicable.

## 3 RESULTS

### 3.1 Treatment planning evaluation

Initial evaluations compared the representation of surrogate markers on SCT and conventional CT images. For fiducial‐based tracking, marker positions were consistent within 1 mm, confirming that the imaging modalities were in close agreement. Due to limitations in the treatment delivery software (which only accepted a 512 × 512 pixel format), a resampling of the original MRI‐only data was required. Although this introduced a minor half‐voxel shift, the issue was resolved by disconnecting the frame‐of‐reference (FOR) during registration.

For lung tracking, treatment planning relied on 4D‐CT with mid‐position reconstructions, which provided reasonable planning geometry given the limitations in jaw tracking range of 12–20 mm in the cranio‐caudal direction. Individual beam gating tolerances were determined in clinical multiprofessional discussions; for prostate cases, these tolerances were uniform due to the consistent anatomical presentation, whereas for lung cases, tolerances were tailored based on the proximity of the target to critical structures such as the medulla or intermediate bronchi.

Plan complexity evaluations revealed that, despite the additional imaging and motion parameters, there was no significant correlation between plan complexity and dosimetric error. Dose calculations on both sCT and conventional CT datasets yielded comparable results in accordance with previously published data (dose difference < 1% within body structure), thus confirming the viability of our planning approach for both fiducial‐based and markerless tracking.

### 3.2 Minimum requirements for real‐time target position monitoring

#### 3.2.1 Geometric accuracy

##### Fiducial tracking

Using clinical prostate motion traces, the Synchrony system improved the geometric accuracy substantially. With tracking enabled, the average RMSE was 0.76 ± 0.27 mm, compared to actual movement of 3.99 ± 2.84 mm when tracking was not applied. Similarly, the MAE was reduced from 1.59 ± 1.27 mm in the absence of tracking to 0.36 ± 0.12 mm with the Synchrony system active (*p* = 0.008). The 95% confidence margin for dynamic prostate tracking was 1.81 mm with tracking and 6.55 mm without. Moreover, the system's predicted potential difference showed an average correlation of 0.49 ± 0.22 with the measured 3D deviation, and the predicted error encompassed the actual error 69% of the time. Summary of results in Table 2.

**TABLE 2:** Summary of selected tests and tolerances from TG‐264.

TG‐264 test performed	Expected tolerance	Measured outcome
Geometric accuracy (fiducial tracking)	RMSE ≤ 1.5 mm	RMSE = 0.76 ± 0.27 mm
Geometric accuracy (markerless tracking)	RMSE ≤ 1.5 mm	RMSE = 0.80 ± 0.15 mm
Dosimetric accuracy (gamma criteria)	GPR ≥ 95% (2%/2 mm)	GPR ≥ 96%
System latency (fiducial tracking)	≤ 500 ms	Approximately 630 ms
Real‐time surrogate accuracy	Accuracy within 1 mm, frequency > 3 Hz	Accuracy within 1 mm, frequency ∼5 Hz

##### Markerless adaptive tracking

**FIGURE 1:**
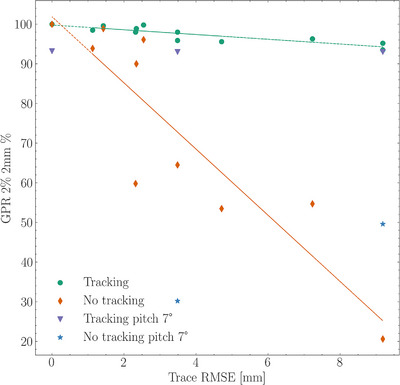
GPR for MLC tracking versus non‐tracking delivery as a function of RMSE of motion trace. Additionally, shown with measurements of plans with a fixed‐pitched phantom position.

Results were similarly promising for adaptive markerless tracking on the dynamic anthropomorphic phantom. Clinical and regular sinusoidal motions produced a mean RMSE of 0.80 ± 0.15 mm and an MAE of 0.68 ± 0.15 mm. In cases with a deliberate 2 mm offset (ZY sinusoidal motion), the RMSE slightly increased to 0.94 mm, while patient‐specific 3D movement yielded an RMSE as low as 0.60 mm. The overall margin of error (95% confidence) was approximately 1.71 ± 0.35 mm, with the best performance observed along the *y*‐axis, figure 1.

#### 3.2.2 Dosimetric accuracy

##### Fiducial tracking

**FIGURE 2:**
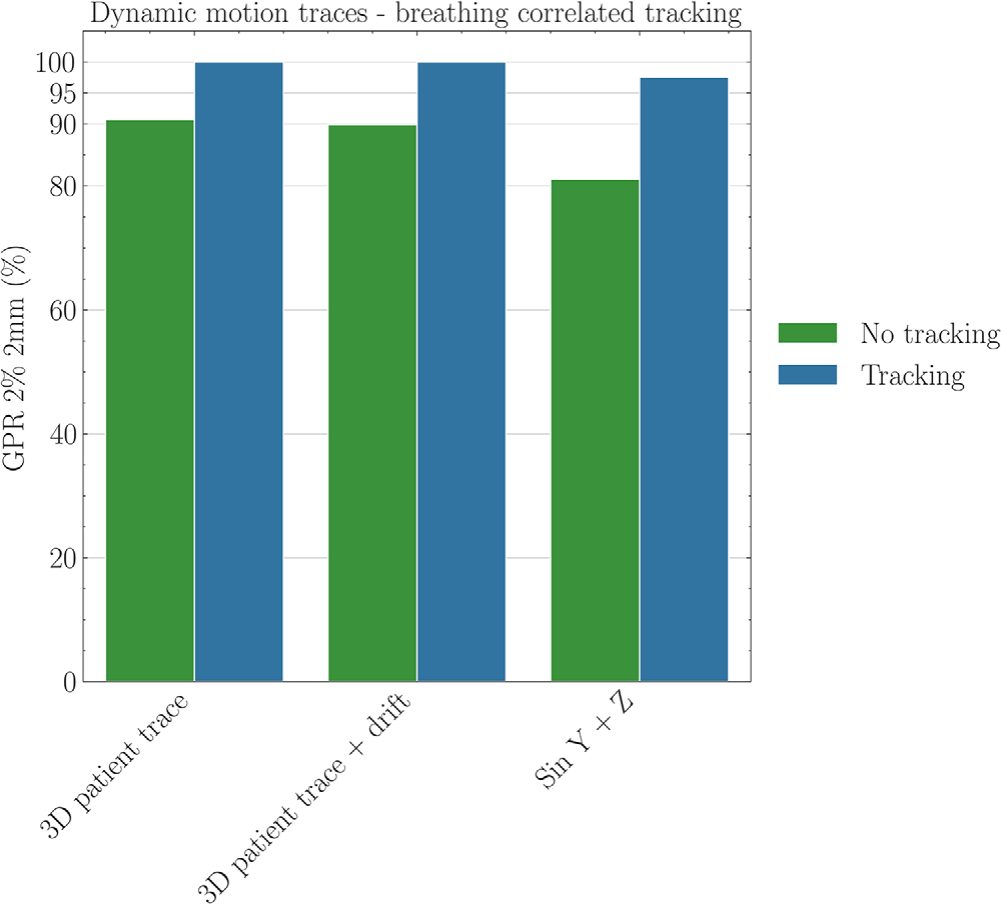
Markerless QA for 3D patient motion traces shown with GPR for patient‐derived motion trace, with/without 5 mm drift of mean position during treatment delivery, and additionally simple sinusoidal motion in Y‐ and Z‐axis with 10 mm amplitude over each axis.

Dosimetric evaluation using gamma analysis (with global criteria of 2% dose difference and 2 mm DTA) showed that Synchrony plans for prostate tracking consistently achieved a 95% GPR, whereas static plans occasionally fell below these thresholds (Figure 2). In tests involving angular deviations (pitch up to 7° and roll up to 5°), the GPR for Synchrony plans remained robust, though a roll of 10°, if not compensated by proper registration, resulted in a lower GPR of 88.3%. When registration protocols were properly adjusted to account for rotational variations by including rotation in the adjustment, the system achieved a 100% GPR within the same tolerance limits. For large static offsets (up to 30 mm), the Synchrony plans maintained acceptable GPRs, whereas non‐adaptive plans were unable to meet clinical criteria, where the difference was significant (*V* = 7.0, *p* = .037).

##### Markerless adaptive tracking

Dosimetric accuracy for markerless tracking was evaluated using both dynamic and static test scenarios. For markerless tracking, the average GPR was 96% (± 5%) using the same gamma criteria, compared to 74% (± 23%) for the same plan without tracking, performed under identical conditions. High‐resolution film dosimetry, using the Quasar phantom with patient‐specific motion trace inputs, confirmed that the delivered dose distribution matched the planned distribution, demonstrating a GPR of 93% (2%, 2 mm).

#### 3.2.3 Other performance metrics and QA

The real‐time surrogate signal was verified to be consistent with log file data, and the overall system latency was measured at approximately 630 ms, from image acquisition to the completion of jaw movement. Although slightly above the ideal 500 ms threshold outlined in TG‐264, this latency remains well within the manufacturer's tolerance of 1.5 s. Additional tests demonstrated that the system could accurately detect and pause treatment in response to periodic motion errors, further supporting the robustness of the MLC tracking algorithm.

A comprehensive end‐to‐end QA program was implemented, integrating data from planning, delivery, and post‐treatment analysis. Patient‐specific QA tests based on the Delta4 system, with an intentional 10 mm offset introduced in certain tests, confirmed that the Synchrony system reliably identified and corrected for the introduced deviations. All initial clinical test plans achieved a GPR of 100%, reinforcing the deliverability of the Synchrony plans.

### 3.3 Safe implementation and clinical workflow

#### 3.3.1 System tolerances and margin considerations

Safe clinical implementation of the MLC tracking system required setting appropriate tolerance thresholds that aligned with TG‐264 guidelines. For fiducial MLC tracking, thresholds were set as follows: T1 (potential difference) at 3 mm, T2 (rigid body) at 3 mm, and T3 (target offset) at 20 mm—though the latter was closely monitored during treatment due to its potential impact on OAR exposure. The auto pause delay (T4) was fixed at 25 s plus the interval to the next kV image, and T5 (target outside jaw range) was set to 0%. For markerless lung tracking, the measured delta (T6) was similarly set to 3 mm.

Initially, CTV to PTV margins were maintained as in non‐tracking treatments. This conservative approach was adopted to ensure that any residual uncertainties did not compromise patient safety. For lung cancer cases, while margins were not explicitly reduced, the lack of a 4D‐based internal target volume (ITV) was noted, and caution was exercised in correlating the reduced margins with patient‐specific respiratory motion patterns. The ESTRO‐ACROP guidelines were also considered to ensure that both inter‐fraction and intra‐fraction errors were appropriately accounted for prior to any margin reduction.

#### 3.3.2 Clinical decision pathways

Clinical decision‐making pathways were developed to guide patient selection and treatment execution. For prostate cancer, inclusion required a confirmed diagnosis of high‐risk disease, eligibility for MRI‐only simulation (i.e., absence of metallic implants), and an ECOG performance status between 0 and 2. Treatment was delivered using hypofractionated radiotherapy with a prescribed dose of 42.7 Gy in seven fractions.

For lung cancer, patients were eligible if they had a confirmed diagnosis of primary or metastatic disease, a clearly distinguishable lesion visible on CT, and the capability for reproducible treatment simulation. In addition, treatments were limited to cases where a safe minimum distance of approximately 1 cm from adjacent critical tissues could be maintained.

During daily treatment, additional checkpoints were applied. In prostate cases, daily online CT images (ClearRT) were scrutinized to confirm that the prostate remained within 1 cm of the simulation CT when registered to bony anatomy, that implanted markers used for final pre‐treatment registration were stable, and that only random (nonperiodic) motion was observed. For lung treatments, similar verification steps ensured that the lesion remained in its expected location and that the adaptive model produced consistent target matches.

#### 3.3.3 Clinical implementation experience

The clinical introduction of the MLC tracking system was preceded by a dedicated training program. Radiation therapists (RTTs) and nurses underwent an initial 2+2 h training session, while medical physicists received an intensive 2 h training session. A radiation oncologist was present during pretreatment setups to provide additional oversight. The implementation was supported by vendor training sessions, supplemented by local commissioning protocols developed by experienced medical physicists.

**FIGURE 3:**
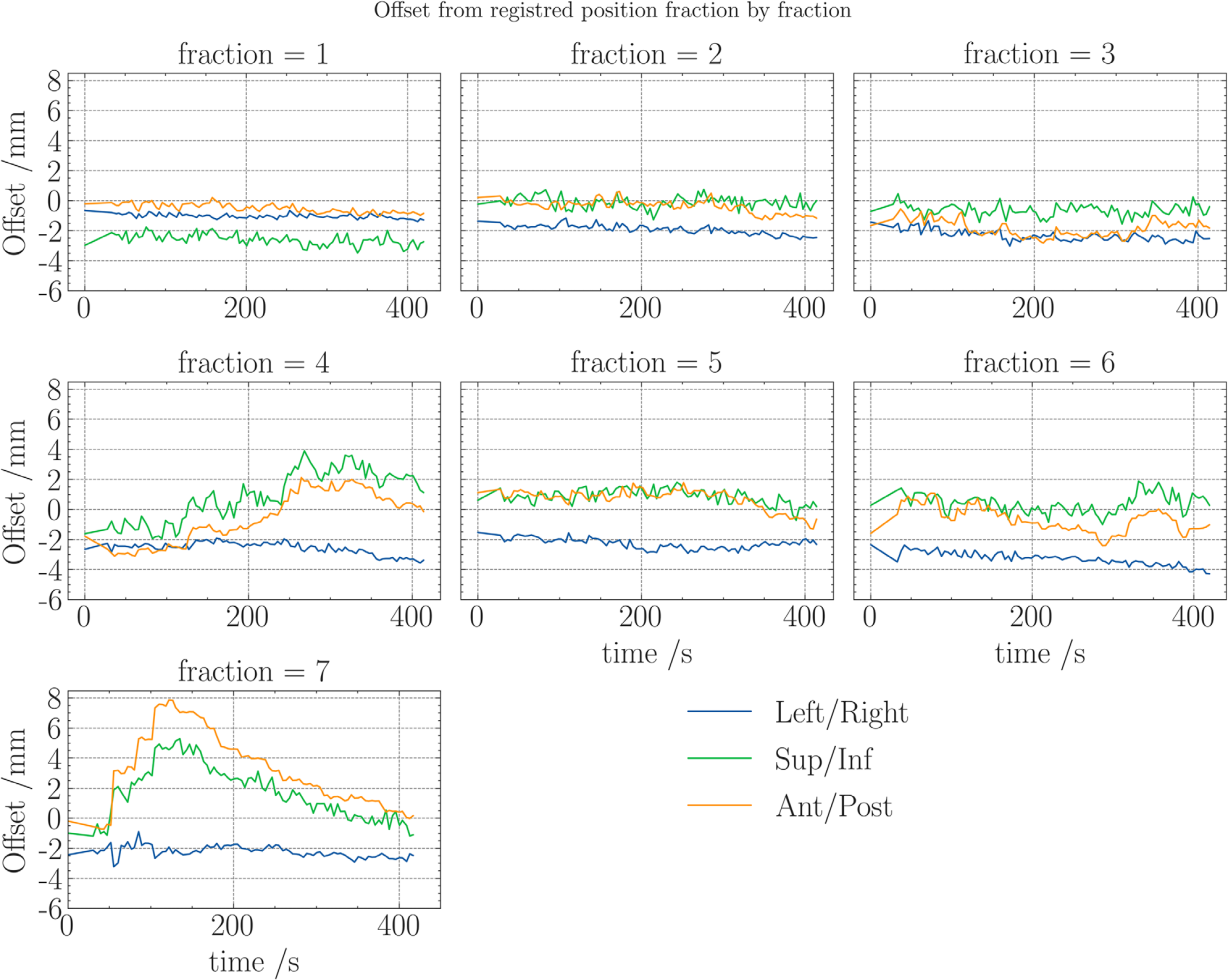
Offset from reference position for each fraction and axis for the first clinical patient. Axes in machine coordinate system superior/inferior (Sup/Inf) with superior in the head direction and thus into the bore, anterior/posterior (Ant/Post) where positive is in the anterior direction. Offset was calculated by the Synchrony system from kV images with 6 kV images per gantry rotation, and a rotation time of 24 s.

**FIGURE 4:**
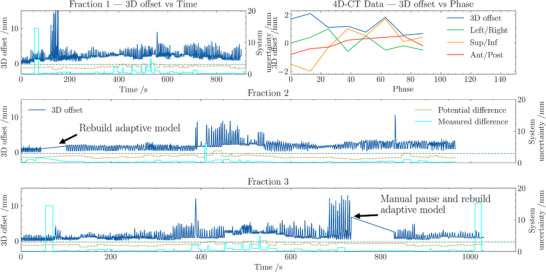
Target offsets for the first lung patient treated with synchrony at our clinic. Offset is registered position for axis left‐right (LR), superior‐inferior (SI) and anterior‐posterior (AP) for treated fraction 1–3.

The initial clinical cases, comprising both a prostate patient using fiducial MLC tracking and a lung patient treated with markerless adaptive tracking, demonstrated the systems feasibility. For the prostate patient, the median 3D deviations from registered offset position across seven fractions ranged from 2.0 to 3.9 mm, with the largest prostate motion noted in the superior and anterior directions (figure 3). Importantly, the daily CT images aligned well with the sCT used for treatment planning, and the use of MLC tracking improved dose coverage significantly in fractions with large prostate motion. In the lung case, the mean 95th percentile deviation was approximately 3.3 mm, and the system's predicted 3D uncertainty was on average 2.1 mm (figure 4). In both instances, the integration of TG‐264 guidelines into clinical practice was shown to enhance treatment safety and efficacy.

## 4 DISCUSSION

In this study, we applied the AAPM TG‐264 guidelines to commission and evaluate an MLC tracking system integrated on a commercial platform, including tumour tracking. Our results show that the system achieves significant improvements in geometric and dosimetric accuracy compared to non‐tracking treatments. With MLC tracking enabled, errors were reduced to levels well within the manufacturer's tolerances, and the dosimetric analysis confirmed that real‐time adjustments resulted in high dose conformity.

Our commissioning process benefited greatly from the structured framework provided by TG‐264. The report's recommendations on treatment planning evaluation, real‐time monitoring, and QA protocols were instrumental in guiding our systematic assessment. However, our experience also highlighted several areas where the TG‐264 guidelines required adaptation. For example, the guidelines were initially developed with C‐arm linacs in mind, and certain aspects, such as the interplay between jaw tracking and MLC tracking on a helical tomotherapy system, necessitated special consideration. The pneumatic, binary nature of the MLC on the system used introduced unique challenges in terms of beam modulation, particularly when the target moved laterally. This problem is caused by the intermittent nature of movement perpendicular to the MLC movement, and target offset less than half the leaf width (leaf width: 6.25 mm at isocenter) is not compensated in that projection. Additionally, the use of an FFF beam required further attention, as the absence of a flattening filter can amplify residual dose errors. This is explained by that any motion from the planned part of the beam, changes the output to the target as the FFF beam profile is not flat. The actual dosimetric impact of these residual errors are not easily quantified, but measurements with tracking but with a target offset less than half a leaf width, and thus not compensated by the tracking system, showed some impact on GPR on the Delta4. Thus, during treatment, online registration can still improve the beam delivery quality despite the use of MLC tracking.

The clinical decision pathways we developed, based on TG‐264 and supplemented by our institutional protocols, allowed us to safely select patients who were most likely to benefit from the MLC tracking system. For prostate cancer, the combination of fiducial tracking and real‐time imaging provided robust assurance of target localization. In lung cancer, where respiratory motion presents a greater challenge, the markerless adaptive tracking mode proved effective in maintaining dosimetric fidelity despite variable breathing patterns.

Our measurements using phantoms simulated both clinical and exaggerated motion patterns. The dosimetry and QA platform used was invaluable for assessing both geometric and dosimetric accuracy under controlled conditions. The close agreement between the measured motion and the predictions from the motion model confirmed the reliability of the system's error estimation. Although our system latency was measured at 630 ms for markerless tracking, slightly above the ideal 500 ms threshold, we consider this acceptable given the overall system performance and the manufacturer's stated tolerance and given that prostate tracking does not necessarily need “real‐time” tracking in contrast to for example lung irradiation. It is important to note that while shorter latencies are desirable, the clinical impact of a 130 ms difference remains to be fully quantified in a real‐world setting. Additionally, for fiducial tracking without breathing monitoring, as the system is reactive and only change tracking point after each image the biggest actual latency is the time between the kV images. The shortest possible interval between images is 2 s.

Dosimetric accuracy was similarly robust. The use of gamma analysis (2%/2 mm criteria), stricter than clinical criteria's (3%/2 mm), provided quantitative evidence that the MLC tracking system maintained high fidelity in dose delivery. For both prostate and lung cases, the tracked plans consistently outperformed static plans, particularly in scenarios with larger target offsets. These findings underscore the clinical value of integrating real‐time tracking into the treatment workflow, especially for targets subject to significant motion. Using a higher pitch in RayStation plans could, in principle, influence modulation and interplay sensitivity. However, our primary comparisons were based on measured geometric and dosimetric performance during delivery (with and without tracking) under matched clinical objectives and similar delivery timing and imaging settings. The sub‐millimeter RMSE values reported here reflect residual tracking error (programmed motion vs logged target offset) under controlled test conditions and should not be interpreted as the magnitude of intra‐fraction motion. Markerless adaptive tracking on the anthropomorphic thorax phantom yielded slightly higher RMSE than some patient‐derived traces, which is expected given increased image‐based localization uncertainty (reduced target contrast, anatomical complexity) and more challenging motion characteristics (e.g., baseline drift and cycle‐to‐cycle variability). Prior studies frequently report tracking accuracy using a variety of endpoints and traces, which can explain differences in calculated RMSE.

Despite these successes, our study also identified several challenges. The application of TG‐264 guidelines in a non‐C‐arm linac setting, such as our helical tomotherapy system, necessitated some modifications to the recommended QA procedures. Additionally, the differences between 2D imager‐based tolerances and the 3D geometry of patient anatomy required careful interpretation. The use of 2D kV projections to estimate 3D target positions meant that small deviations on single images might occur due to larger deviations along the imaging axis. To mitigate this, we based our interpretation on reconstructed 3D offsets across all projections, verified the correspondence in phantom tests, and maintained conservative margins during clinical implementation. Additionally, the 2D tolerances were compared to offsets in 3D, to assess the practical use of the different tolerances. Furthermore, the relationship between system tolerances (defined in the TG‐264 guidelines) and clinical margin reductions is complex. While it is tempting to reduce PTV margins in the presence of robust MLC tracking, our experience reinforced the caution expressed in TG‐264: premature margin reduction could risk underdosing the target, especially if unforeseen residual errors occur.

A further consideration is the educational aspect. Implementing an online adaptive tracking system with multiple modes and numerous parameters demands a high level of expertise from the clinical team. Our training program, which included hands‐on sessions and vendor‐led workshops, was critical to ensuring that all staff members could operate the system effectively. Ongoing education and periodic retraining was necessary to keep pace with software updates and evolving clinical practices.

Our study also led us to consider the need for a dedicated clinical trial to evaluate margin reduction strategies. Although our initial results with standard margins were encouraging, a formal investigation is required to determine whether reduced margins can be safely implemented without compromising target coverage. Such trials would not only validate our current approach but also contribute valuable data to further refine the TG‐264 guideline and similar efforts. With this as an inspiration, the REMIND trial has been initiated by our center and is currently recruiting patients (ClinicalTrials.gov ID: NCT05844761)^19^

Our results are largely consistent with those reported in previous clinical implementations of the Radixact Synchrony system. For example, Chen et al. (2021)^7^ described initial clinical experiences with Synchrony for lung and prostate cancer, highlighting geometric tracking accuracies within 1–2 mm and gamma pass rates above 95% using typical clinical criteria (2%/2 mm). Our findings align closely with these benchmarks, achieving similar geometric accuracy and dosimetric fidelity during phantom and patient‐specific testing. Furthermore, Goddard et al. (2022)^9^ emphasized the importance of rigorous quality assurance protocols, noting that daily and monthly verification tests are critical to maintaining optimal performance. Our implementation similarly underscores robust and frequent QA as recommended by TG‐264 guidelines. In our study, we tested the fiducial tracking system latency, and previous studies have verified the adaptive lung tracking latency to well within tolerances^6^ Additionally, unlike Ferris et al. (2020)^8^ who highlighted significant modelling challenges associated with variable breathing patterns in markerless adaptive tracking, our markerless tracking evaluations demonstrated consistent performance even with patient‐specific respiratory motion traces. This difference might reflect variations in the adaptive modelling techniques employed or the patient‐specific motion complexity studied. Overall, our work confirms and expands upon earlier findings, reinforcing the importance of adapting TG‐264 guidelines practically to the specific hardware characteristics and clinical contexts of individual radiotherapy centers.

## 5 CONCLUSIONS

This study represents the first reported clinical evaluation of an MLC tracking system using the AAPM TG‐264 guidelines. Overall, we were able to implement the TG‐264 recommendations successfully, achieving significant improvements in geometric and dosimetric accuracy. The integration of fiducial‐based and markerless adaptive tracking modes on the Radixact system demonstrated that real‐time motion compensation can be achieved reliably, even in the presence of complex intra‐fractional motion.

However, our experience also highlighted several challenges inherent in adapting TG‐264 guidelines to non‐standard platforms. The unique characteristics of the pneumatic MLC, jaw tracking for longitudinal motion, and the use of FFF beams required specific modifications to the standard QA and treatment planning procedures. In addition, the relationship between system tolerances and clinical margins remains complex, underscoring the need for further investigation, ideally through a dedicated clinical trial focused on margin reduction.

In conclusion, while the TG‐264 guidelines provide a robust framework for the safe implementation of motion tracking systems, practical experience reveals the need for ongoing refinement and adaptation. Our findings support the clinical value of MLC tracking and emphasize the importance of rigorous QA, targeted staff training, and a conservative approach to margin reduction during the early phases of implementation.

## AUTHOR CONTRIBUTIONS

AH: Idea, Theory, experiment, draft/writing, funding, PI clinical study. TP: Support theory development, experiment, draft feedback. EP: Support theory development, draft feedback. HE: experiment, draft feedback. MK: experiment, draft feedback. AG: Support theory development, draft feedback, supervision medical. PMR: Support theory development, draft feedback, supervision

## CONFLICT OF INTEREST STATEMENT

The authors all declare no conflict of interest.
